# Influence of cigarette smoking on drugs’ metabolism and effects: a systematic review

**DOI:** 10.1007/s00228-025-03817-7

**Published:** 2025-03-20

**Authors:** Stefano Zanni, Jole Del Prete, Alessandra Capogrossi, Giuseppe Papapietro, Angela Del Cimmuto, Sergio Gazzanelli, Andrea Caronna, Carmela Protano

**Affiliations:** Department of Public Health and Infectious Diseases, Sapienza University of Rome, P.Le Aldo Moro 5, 00185 Rome, Italy

**Keywords:** Smoking, Drug, Pharmacokinetics, Pharmacodynamics, Systematic review

## Abstract

**Purpose:**

Cigarette smoke continues to be widely used around the world and it contains several substances that can affect the pharmacokinetics and/or pharmacodynamics of medications, altering their safety and effectiveness. The aim of this systematic review was to summarize the scientific evidence regarding possible changes in the pharmacokinetics and/or pharmacodynamics of drugs induced by cigarette smoking, possible mechanisms of action and related effects.

**Methods:**

The systematic review was performed according to the PRISMA Statement and the protocol was registered on the PROSPERO platform (CRD42023477784). Pubmed, Scopus, Web of Science databases were used. We considered observational, semi-experimental or experimental studies written in English and published between January 1, 2000, and November 13, 2024, focused on smoking subjects (healthy volunteers or patients) receiving any kind of medication. Data regarding possible modifications in drugs’ pharmacokinetics and/or pharmacodynamics induced by cigarette smoking were assessed. The quality of observational studies and experimental studies was evaluated using the Newcastle–Ottawa Quality Assessment Scale and the Jadad Scale, respectively.

**Results:**

In total, 37 studies were included, and 31 of them showed relevant modifications in the pharmacokinetics or effects of the drugs in smokers compared to non-smokers. Most of the included studies (*n* = 20) investigated drugs for psychiatric or neurological disorders, showing a reduction in plasma concentration or an increase in drug clearance in smokers as well as antibiotics metronidazole and cycloserine. Besides, seven articles focused on anticancer drugs indicating an increase in drug metabolism. The remaining articles reported effects of smoking on the metabolism of other drugs, such as cardiovascular drugs, phosphodiesterase 5 inhibitors, local anesthetics and medications for musculoskeletal or chronic obstructive pulmonary diseases. Induction of the cytochrome enzyme CYP1A2 is the most common mechanism mediating the reduction of drug concentrations by cigarette smoking.

**Conclusion:**

The results indicate an increased risk of therapeutic failure for smokers and represent further motivation to encourage smoking cessation or attention in formulating personalized therapy.

## Introduction

Tobacco use continues to be one of the biggest public health threats, accounting for over 8 million tobacco-related deaths per years, and tobacco market supervision remains a global health priority [1].

The adverse health effects associated with traditional smoking have long been known and they have related to the thousands chemicals present in smoke, most of which are toxic, carcinogenic, and mutagenic. Indeed, scientific evidence has shown that smoking traditional cigarettes can determine chronic obstructive pulmonary diseases [2], interstitial lung diseases [3], cardiovascular diseases [4, 5], atherosclerosis [6], cerebrovascular strokes [7, 8], neurodegenerative diseases [9] such as Alzheimer’s disease [10], multiple sclerosis [11], and various kind of cancers [12]. Besides, exposure to tobacco smoke—both active and passive exposure to traditional cigarettes—during pregnancy can cause negative maternal and neonatal outcomes, such as infertility, low birth weight, preterm birth, antepartum and intrapartum stillbirth and perinatal birth [13]. Further scientific evidence in this field has highlighted that smoking traditional cigarette can modify the therapeutic activity of a great number of drugs [14]. In particular, some substances present in traditional cigarette smoke, such as polycyclic aromatic hydrocarbons (PAHs), acetone, pyridine, heavy metals, benzene, carbon monoxide, nicotine, can influence absorption, distribution, metabolism and elimination of a drug and it can alter the response of the cellular receptors on which they act, resulting in changes of the effectiveness and safety of pharmacological treatments. The exact mechanism underlying these effects is still unclear. However, an important phenomenon implicated seems to be the inhibition and/or induction of isozymes of the cytochrome P450 (CYP) family among smokers [15]. In order to understand this hypothesis, it must be considered that drugs are mainly metabolized in the liver through two metabolism phases: phase I and phase II. Phase I modifications, which include oxidation, reduction, hydrolysis, cyclization/decyclization and removal of hydrogen or addition of oxygen to more polar molecules, result in changes in the chemical structure of the lipophilic drug. Most phase I reactions are catalyzed by the cytochrome P450 system, also known as microsomal mixed-function oxidase. Sometimes, in this process, an inactive pro-drug is transformed into a metabolically active drug. Processes in this phase often produce metabolites that still retain some of their pharmacological activity and may become substrates of phase II reactions. Phase II modifications, which include methylation, acetylation, sulphation, glucuronidation and conjugation with glycine or glutathione, are conjugation reactions. These reactions render the compounds pharmacologically inert and water-soluble in order to be easily excreted. [16, 17]. One of the main players involved in the drug metabolism are the hepatic cytochromes, involving numerous families and subfamilies of enzymes which catalyze these processes [16]. Indeed, the isoenzymes of the CYP family are those most involved in drug metabolism, like CYP2C9 (ibuprofen), CYP2C19 (omeprazole), CYP2D6 (propranolol), CYP2E1 (verapamil), CYP3A4 (clopidogrel), and CYP1A2 (clozapine) [18]. This last cytochrome isoform, for example, plays a key role in the metabolism of widely used drugs such as theophylline, lidocaine and clozapine, but it also intervenes in the activation of certain pro-carcinogens [20]. Since the activity of CYP1A2 varies greatly from individual to individual, literature evidenced that there are different influential factors, such as genetics, coffee consumption, oral contraceptive use and smoking, even if the mechanisms are complex and not completely clear [21]. Another family of enzymes responsible for drug metabolism, particularly in phase 2 reactions, is the UDP-glucuronosyltransferase (UGT) family [22]. For example, the UGT1A4 isoform is involved in clozapine metabolism [23].

We focused the present systematic review on smoking. It is known that tobacco smoke can interact with drugs both through pharmacokinetic and pharmacodynamic mechanisms [24]. The scientific literature has shown that some chemicals produced by tobacco combustion, such as PAHs, are potent enzyme inducers of the CYP1A2 and UGT isoforms [15, 18]. Several studies reported that the induction of CYP1A2 determined by smoking cigarette can result in a reduction of plasma levels of drugs that are mainly metabolized by this isoenzyme, with a decrease in therapeutic response [24–27]. Conversely, smoking cessation would lead to a reduction in CYP1A2 activity [28] suggesting that, in ex-smoking patients, maintaining the same therapeutic dosages of certain drugs can lead to an increase in plasma levels, with a possible risk of toxicity. The increase in plasma levels of certain drugs has also been observed in smoking patients who have switched from traditional cigarettes to electronic cigarettes. Indeed, a case report showed that the serum levels of clozapine did not change according to expectations in a smoking patient who switched from traditional cigarettes to electronic ones, determining, probably, the arrest of the induction of the CYP1A2 enzyme [29].

Besides, cigarette smoking seems to acts as a suppressor on the function of P-glycoprotein (P-gp), which participates in the export of numerous toxins, drugs, and physiological compounds [30].

The influence of cigarette smoking on drugs’ effects should be carefully taken into consideration because scientific progress is increasingly turned to a personalized medicine by assessing all aspects that may lead or not to therapeutic success. Thus, dosage readjustment and therapeutic monitoring of therapies in patients who smoke, starting or stopping smoking would be indicated [25, 31]. Besides, it is essential to investigate which drugs and mechanisms of action are involved and which effects occur [32]. Research in this field could help improve the effectiveness of treatments, taking into account individual variations due to factors such as smoking [33].

The aim of this systematic review was to summarize the scientific evidence regarding possible changes in the pharmacokinetics and/or pharmacodynamics of drugs induced by cigarette smoking, possible mechanisms of action and related effects.

## Materials and methods

### Search strategy and selection procedures

The systematic review was conducted following the guidelines PRISMA (Preferred Reporting Items for Systematic Reviews and Meta-Analyses) [34]. Moreover, the review protocol was registered in PROSPERO (reference number CRD42023477784).

The bibliographic and citation databases that we used were PubMed, Web of Science (Science and Social Science Citation Index), and Scopus. The following query was used for the search: (“smoking” OR “tobacco”) AND (“drug Interactions”) AND (“pharmacodynamics” OR “pharmacokinetics”). The search was conducted from November 9th, 2024, until November 13, 2024.

### PICOs statement

To develop the research question for identifying studies to include in the review, the “PICO(S)” methodology was employed, where “P” represents patient, population, or problem; “I” denotes intervention; “C” refers to the control group or comparison; “O” for outcome, and “S” for study design. The selected population included cigarette smoking patients or healthy cigarette smoking volunteers of all age and gender. The intervention considered was any type of regularly prescribed pharmaceutical therapy or healthy volunteers taking the pharmaceutical drug. The comparison control group was matched by age, gender, and condition of non-smoker patients or volunteers (if present), or smokers with a lower daily cigarette consumption. The outcome was any differences in pharmacokinetics or pharmacodynamics of drugs observed among smokers compared to non-smokers or lighter smokers.

### Inclusion and exclusion criteria

Articles were considered eligible if they presented data from observational or semi-experimental or experimental studies on smoking patients receiving any pharmaceutical therapy or healthy volunteer smokers receiving the therapy, regardless of gender or age, published in English language from January 1, 2000, to November 13, 2024.

Studies about illegal drugs or smoking cessation medications, or that did not involve human subjects such as in vitro and animal experimental studies, or with control group with different socio-demographic characteristics were excluded. Besides, all articles not reporting original data, such as reviews, systematic reviews, case studies, proceedings, qualitative investigations, book chapters, editorials, and commentary studies were also excluded.

The abstracts and title pages collected from the databases have been imported into the bibliographic management program Zotero, which was utilized for preliminary relevance evaluation. Following that, a title and abstract screening was performed, with possibly suitable papers being separately examined by four authors (S.Z., J.D.P., A.Ci, G.P.). Following that, the full texts of eligible articles were reviewed independently by the same four authors, and a discussion ensued on their possible insertion in the review was performed. Any emerged issues were addressed through author consensus.

### Data extraction process and quality assessment

The information gathered was synthetized in a table that included bibliographic information such as author, publication year, origin country, and study design information as well as population sample details such as sample size, age, and gender of participants. The data table additionally provides details regarding the comparison group, smoking habits (if assessed), the pharmaceutical molecule studied, a description of the intervention and its outcomes, the postulated pharmacokinetic and pharmacodynamic mechanisms, the enzymes involved in the metabolism of drugs and affected by smoking, studied in each included article and the major findings for each study included.

The Newcastle–Ottawa Quality Assessment Scale, adapted for cohort, case–control, and cross-sectional studies, was used to assess the quality of observational studies, while the Jadad Scale was used to assess the methodological quality of randomized clinical trials. The overall rating was calculated using these scales. Each study was rated individually by four authors (S.Z., J.D.P., A.Ci, G.P.), and any disagreements were handled through a discussion among all authors. The mean of the authors’ scores was used to calculate the final rating for each article.

## Results

**Fig. 1:**
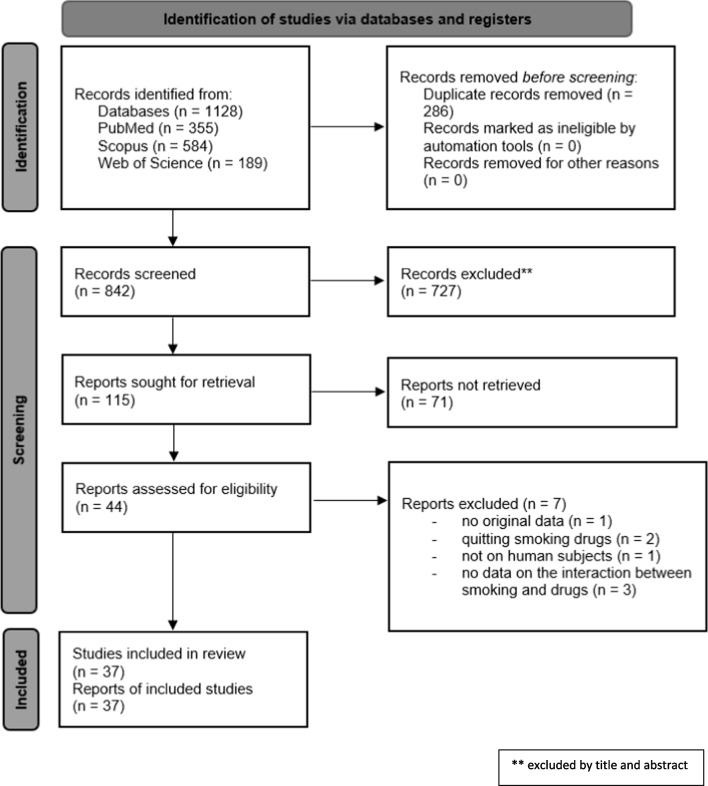
Flow chart describing the research process

The details of the review process are reported in Fig. 1.

The bibliographic search in the three databases used produced 1128 titles (355 from PubMed, 584 from Scopus, and 189 from Web of Science). After removing duplicates, 842 records were obtained. During the first screening by title and abstract, 727 articles were removed. Following the reading of the remaining 115 articles, 71 were further excluded, as they did not match the inclusion criteria established for this review. Additionally, we eliminated one article lacking original data, two articles because were related to smoking cessation medications, one study because it was not conducted on human subjects, and three studies because they did not present data on the interaction between smoking and pharmaceuticals. At the conclusion of the selection process, 37 studies were included in the review.

**Fig. 2:**
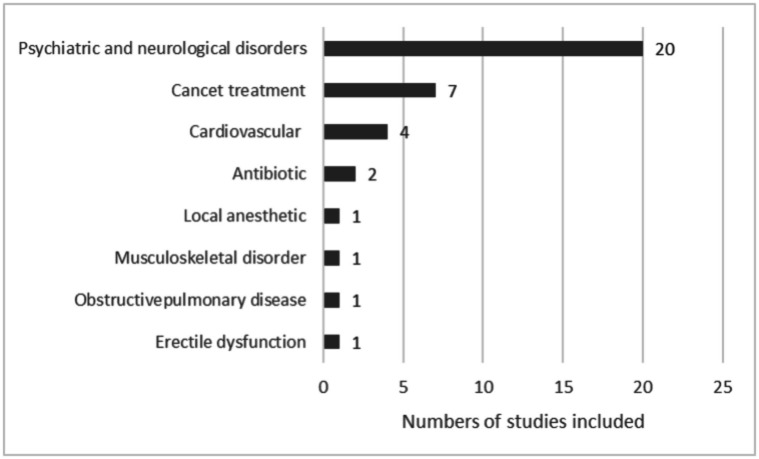
Types of pharmaceuticals or disease treated with pharmaceuticals studied by the included articles

Figure 2 reports the types of pharmaceuticals or disease type treated with pharmaceuticals studied by the included studies.

As shown in Fig. 2, most of the articles involved psychiatric drugs, followed by cancer treatment and cardiovascular medications.

The results of the included studies and their main characteristics are shown in Table 1.

**Table 1:** Main characteristics and results of the studies included in the review

Author, year, country; study design	Sample size and study population; mean age in years (± SD and/or range), gender; comparison studied pharmaceuticals and type	Intervention	Outcomes	Enzymes involved in the metabolism of drugs studied in the clinical trial	Results	Quality of included studies
Jokinen [35], 2001, Finland; cohort	18 healthy volunteers, 8 smokers, 10 non-smokers; non-smokers 23 (± 3), smokers 25 (± 4), 9 males, 9 females; non-smoker volunteers Ropivacaine; local anesthetic	In both groups, each subject ingested daily for 5 days either placebo or 600 mg rifampin. On day 6 each subject received intravenously over 30 min a single dose of 0.6 mg/kg ropivacaine	Ropivacaine in venous plasma and urine were measured for up to 12 h and 24 h, respectively. The area under the ropivacaine plasma concentration–time curve was estimated by means of the logarithmic trapezoidal rule with extrapolation to infinity [AUC(O- ∞)]. Elimination half-lives (t) were calculated from the following equation: t = ln2/k. The plasma clearance (CL) of ropivacaine was computed as CL = Dose/AUC, and renal clearance (CLR) was CL, = f x CL	CYP 1A2 CYP 3A4	There were no statistically significant differences in the area under the plasma concentration–time curve (AUC), plasma clearance (CL), or half-life (t) of ropivacaine between the smokers and non-smokers. However, smokers excreted in urine 31% more 3-OH-ropivacaine and 62% less PPX than non-smokers did. Rifampin decreased the AUC of ropivacaine in non-smokers by 52% and in smokers by 38%	Fair
Carrillo [36] 2002, Spain; cohort	17 psychiatric patients, 8 smokers, 9 non-smokers; 36.9 (± 13), 8 males, 9 females; non-smokers psychiatric patients Olanzapine; psychiatric and neurological disorders	All patients received monotherapy, for 15 days, with a stable single dose of olanzapine at a mean dosage of 10 mg/day for all smokers and 7.5 ± 2.5 mg/day (range, 5–10 mg) for non-smokers	Olanzapine C:D (Plasmatic concentration/Dose) ratio was calculated by dividing the steady-state plasma concentration of the antipsychotic drug in nanograms per milliliter by the olanzapine daily dose in milligrams. Percentage decrease in brief Psychiatric Rating Scale (BPRS) total score after 15 days of therapy	CYP1A2	The olanzapine plasma C:D ratio (ng/mL/mg) was about fivefold lower in smokers (7.9 ± 2.6) than in non-smokers (1.56 ± 1.1; *p* < 0.0001). After day 15 of the antipsychotic therapy, the mean percentage decrease in brief BPRS total score relative to the predosing score (in the drug-free period) was higher for non-smokers than for smokers (30.4 ± 10% vs. 12.5 ± 14%; *p* < 0.01). This measure of olanzapine effectiveness was higher in non-smokers (*p* < 0.01) than in smokers. Smoking-induced increased CYP1A2 activity significantly diminished plasma olanzapine concentrations and the antipsychotic effect of the drug	Good
Fuhr [37], 2002, Germany; cohort	24 healthy patients, 12 smokers, 12 non-smokers; smokers: 27 (± 3), 6 males, 6 females non-smokers: 27 (± 4), 6 males, 6 females non-smokers Verapamil; cardiovascular	Isoptin KHK (Knoll AG, Ludwigshafen, Germany), a prolonged release preparation containing 120 mg verapamil, was administered every 12 h in two periods of 7 days each. The periods with administration of the study drug followed each other immediately and were not separated by a wash out period	Pharmacokinetic characteristics of verapamil and norverapamil	CYP 1A2 CYP 3A4	Smokers had significantly lower AUC and Cmax values than non-smokers by (means) 0.61-fold to 0.85-fold for verapamil and norverapamil enantiomers, respectively	Good
Olubodun [38], 2002, USA.; cohort	16, 5 smokers, 11 non-smokers; 27.8 ± 5.3, 0 males, 16 females; non-smokers Zolpidem; psychiatric and neurological disorders	After overnight fasting and following a predose baseline blood sample, 5 mg of zolpidem tartrate (containing 4.2 mg of zolpidem base) was administered orally with water	Pharmacokinetic characteristics of zolpidem	CYP 1A2 CYP 1A1 CYP 3A4	Clearance was higher (445 vs. 345 ml/min) and half-life was shorter (1.8 vs. 2.7 h) in smokers than non-smokers, although the differences were not statistically significant	Fair
Gex-Fabry [39], 2003, Switzerland; cross-sectional	250 patients, 70 smokers, 180 non-smokers; 123 males, 127 females; non smokers Olanzapine; psychiatric and neurological disorders	Therapeutic drug monitoring (TDM) data for the antipsychotic drug olanzapine were investigated. The study included 250 patients who received olanzapine orally and had concentration measured after at least 7 days of treatment on a constant dosing regimen	Daily dose, olanzapine concentration, and concentration to-daily dose ratio (C/D)	CYP1A2	Smokers displayed significantly reduced concentration compared with non-smokers (− 12%). When the different predictors were considered together, the proposed multiplicative effects model predicted 60% higher concentration in elderly, non-smoking women than in young smoking men	Fair
Murdoch [40], 2004, Germany; cohort	25 patients, 12 smokers, 13 non-smokers; 18–65 (± 5 years), 25 males, 0 females; non-smoker patients Cilomilast; obstructive pulmonary disease	A single 15-mg oral dose of cilomilast to either smokers or non-smokers	Determination of cilomilast plasma concentrations and pharmacokinetic profiles such as Cmax (μg/mL), AUC0-∞ (μg•h/mL), t max(h), t1/2(h)	CYP1A2	On comparing the pharmacokinetic profile of cilomilast between smokers and non-smokers, no clinically important differences were observed. On average, values of AUC0-∞ and Cmax were approximately 10% and 19% lower, respectively, in smokers compared with non-smokers. The half-life (t1/2) of cilomilast was similar in smokers and non-smokers. Taken together with AUC0-∞, no differences in the clearance of cilomilast due to smoking were noted. Smoking had little effect on the pharmacokinetic profile of cilomilast, indicating an absence of a dose adjustment requirement in smokers	Good
Diaz [41], 2005, USA; cohort	90 psychiatric patients, 74 smokers, 14 non-smokers, 2 not assessed for smoking status; 45 (± 10), 42 males, 48 females; heavy smokers versus non-heavy smokers Clozapine psychiatric and neurological disorders	47 patients randomized to 100, 300, or 600 mg/day for 16-week double-blind clozapine trials	Plasma clozapine and norclozapine concentrations, the mean coefficient of variation (CV) of plasma clozapine concentrations	CYP1A2	Under 100 mg/day, the mean CV for clozapine concentrations was significantly higher for heavy smokers than non-heavy smokers (32%, SD = 3 vs. 19%, SD = 8) (*p* = 0.03)	Good
Hamilton [42], 2006, USA; cohort	32 patients, 16 smokers, 16 non-smokers; non-smokers 31 (19–52), smokers 39 (19–54), 32 males, 0 females; non-smoker patients erlotinib; cancer treatment	All subjects were to receive a single dose of 150 mg erlotinib on day 1 followed by a single dose of 300 mg erlotinib on day 15	The pharmacokinetic variables included Cmax, Tmax, C24h, AUC_0-∞_ (area under the curve, and T1/2Ez	CYP1A2 CYP3A4 CYP2C8	Current smokers achieved significantly less erlotinib exposure following a single 150 or 300 mg dose than non-smokers. Following the 150 mg dose, the geometric mean erlotinib AUC_0-∞_ (area under the curve) in smokers was 2.8-fold lower than in non-smokers and similar to that of non-smokers at the 300 mg dose. Cmax in smokers was two-thirds of that in non-smokers, and C24h in smokers was 8.3-fold lower than in non-smokers. The median C24h of smokers at the 300 mg dose was slightly less than the C24h of smokers at the 150 mg dose. The median Cmax was greater in smokers at the 300 mg dose than in non-smokers at the 150 mg dose	Good
Haslemo [43], 2006, Norway; cohort	73 schizophrenic patients recruited from psychiatric nursing homes, 59 smokers, 14 non-smokers; 52 (± 9), 50 males, 23 females; non-smoker patients	The patients received long-term treatment with clozapine (n = 33) or olanzapine (n = 40), on average, the last 18 months before the study. Doses had been adjusted to individual requirements, but were unchanged for at least 4 weeks prior to the study	Clozapine and olanzapine C/D (Plasmatic concentration/Dose) ratios in patients with different smoking habits	CYP1A2	While the mean ratio was twice as high in non-smokers compared to smokers for both drugs (*p* < 0.01), the C/D ratios of clozapine and olanzapine were not significantly different between the subgroups of smokers (p > 0.15). Absolute serum concentrations were also higher in non-smokers compared to smokers: 50% for clozapine (*p* = 0.058) and 67% for olanzapine (*p* < 0.01). A daily consumption of 7–12 cigarettes is probably sufficient for maximum induction of clozapine and olanzapine metabolism	Good
Kim [44], 2006, USA; cohort	18 healthy volunteers, 9 smokers, 9 non-smokers; 39.8 ± 10.2, 9 males, 9 females; non-smokers S-warfarin; cardiovascular	A randomized, single dose, two-treatment crossover study of warfarin with a washout period of 21 days was performed	AUC of the S-warfarin enantiomer	CYP2C9	The S-warfarin AUC between smokers and non-smokers did not differ by > 25% after inhibition. There was no difference in S-warfarin AUC during baseline (*p* = 0.45) or inhibition (*p* = 0.19) periods for smokers versus non-smokers. Cigarette smoking did not affect CYP2C9 activity	Poor
Theisen [45], 2006, Germany; cross-sectional	122 patients of the Department of Child and Adolescent Psychiatry, University of Marburg, Germany, 48 smokers, 74 non-smokers; 16.9 ± 2.2, 74 males, 48 females; non-smoker patients Olanzapine (OLZ) and its metabolites N-desmethyl OLZ (DMO) and 2- hydroxymethyl OLZ (2-OH-OLZ); psychiatric and neurological disorders	Data about patients that were prescribed OLZ orally, between 1998 and June 2005, collected from routine therapeutic drug monitoring (TDM) at the Department of Child and Adolescent Psychiatry	Serum concentrations, and concentration-to-dose (C/D) ratios of OLZ and its metabolites, N-desmethyl OLZ (DMO), and 2-hydroxymethyl OLZ (2-OH-OLZ)	CYP1A2 CYP2D6	Smokers exhibited significantly lower C/D ratios for OLZ (*p* = 0.008), DMO (*p* = 0.001), and 2-OH-OLZ (*p* = 0.021), respectively, than non-smokers. Smokers did not get higher (*p* = 0.088) prescribed doses than non-smokers, even when grouped by sex	Good
Backman [46], 2007, Finland; cross-sectional	71 healthy volunteers, 15 smokers, 36 non-smokers; 23 (19–31), 53 males, 18 females; non-smoker volunteers Tizanidine; musculoskeletal disorders	Each subject ingested a single oral dose of 4 mg of tizanidine; an oral caffeine test was performed one day before tizanidine administration, with 100 mg of caffeine	For the Tizanidine, it was estimates the peak of concentration in plasma (Cmax), time to Cmax (tmax), AUC(0-∞), and elimination half-life (t1/2). Systolic and diastolic blood pressures, subjective drowsiness, subjective overall drug effect, and the Digit Symbol Substitution Test (DSST) were assessed before administration oftizanidine and immediately after each blood sampling	CYP1A2	The mean plasma concentrations of tizanidine were lower in male smokers than in non-smokers. The AUC(0-∞) of tizanidine was 38% smaller in male smokers than in male non-smokers, but the Cmax was not statistically significantly lower in male smokers than in male non-smokers. However, both the Cmax and AUC (0-∞) of tizanidine were considerably lower in smoking men than in non-smoking women. The t1/2 was 10% shorter in male smokers than in male non-smokers	Poor
Van der Bol [47], 2007, USA; cohort	190 patients, 49 smokers, 141 non-smokers; 110 males, 80 females; non-smoker patients Irinotecan; cancer treatment	Patients received irinotecan once every 3 weeks as a 90-min intravenous infusion at doses ranging from 175 to 350 mg/m^2^ or a 600-mg flat-fixed dose	Pharmacokinetic characteristics of Irinotecan	CYP 1A2 CYP 1A1 CYP 3A4	In smokers, the dose-normalized area under the plasma concentration–time curve of irinotecan was significantly lower (median, 28.7 v 33.9 ng h/mL/mg; *p* < 0.001) compared with non-smokers. smokers showed an almost 40% lower exposure to SN-38 (median, 0.54 v 0.87 ng ·h/mL/mg; *p* < 0.001) and a higher relative extent of glucuronidation of SN-38 into SN-38G (median, 6.6 v 4.5; *p* < 0.006). Smokers experienced considerably less hematologic toxicity. In particular, the incidence of grade 3 to 4 neutropenia was 6% in smokers versus 38% in non-smokers	Good
Van Erp [48], 2008, Europe; cross-sectional	45 patients, 15 smokers, 30 non-smokers; 51.3 (21.0–69.9), 28 males, 15 females; non-smoker patients imatinib; cancer treatment	Patients were treated with imatinib 400 mg once daily (7 patients), 300 mg twice daily (7 patients), 400 mg twice daily (25patients), or 500 mg twice daily (6 patients)	All toxicities were graded using the National Cancer Institute Common Toxicity Criteria version 2.0	CYP 1A2 CYP 3A4	The imatinib exposure in smokers versus non-smokers was not significantly different. This study suggests that the pharmacokinetics of imatinib is not affected by smoking	Good
Yousef [49], 2008, Jordan; cohort	76, 27 smokers, 49 non-smokers; 29.1 ± 6.89, 76 males, 0 females; non-smokers Clopidogrel; cardiovascular	Healthy adult male volunteers were selected randomly. Each subject received a single 75 mg oral dose of clopidogrel after overnight fast	Clopidogrel carboxylate plasma levels were measured and non-compartmental analysis was used to determine peak plasma concentration (Cmax), time to peak plasma concentration (Tmax), elimination half-life (t1/2e), and area under the curve (AUC)	CYP 1A2 CYP 2C19 CYP3A4 CYP 3A5	Smoking is a significant factor affecting the pharmacokinetics of clopidogrel. Smokers had lower AUC and shorter half-life	Good
Ng [50], 2009, Canada; cross-sectional	519 samples from 197 patients treated with clozapine, 98 smokers, 81 non-smokers, 18 not known; 38 (± 13), 138 males, 59 females; non-smokers Clozapine; psychiatric and neurological disorders	Analysis of data already collected from inpatients with any psychiatric disorder across all Centre for Addiction and Mental Health sites during routine TDM from 2001 to 2007	Pharmacokinetic characteristics of clozapine and norclozapine	CYP 1A2	For clozapine, smokers showed increased oral clearance by 6.0 L/h. For norclozapine, smokers were associated with an increased oral clearance of 11.3 L/h	Fair
Cormac [51], 2010, United Kingdom; cohort	51 psychiatric inpatients taking clozapine, 48 smokers, 3 non-smokers; NA; plasmatic levels after smoking cessation clozapine; psychiatric and neurological disorders	Retrospective data on clozapine dose and plasma levels were collected from a 3-month period before and a 6-month period after the introduction of the smoking ban	Clozapine plasma concentration	CYP 1A2	Before the ban only 4.2% of patients who smoked had a plasma clozapine level ± 1000 μg/l but after the ban this increased to 41.7% of the sample within the 6-month period following the ban despite dose reductions	Poor
Patel [52], 2011, UK; cross-sectional	627 samples from adult patients over 3207 patients; NA; non-smokers Olanzapine; psychiatric and neurological disorders	Audited data arising from the analysis of blood samples (use of EDTA anticoagulant requested) submitted for plasma olanzapine assay (olanzapine TDM) from patients from the UK and Eire, 1999–2009	Olanzapine plasma concentration	CYP 1A2	The plasma olanzapine was increased by 47% in female non-smokers compared with female smokers and 59% in male non-smokers compared with male smokers	Fair
Bowskill [53], 2012, United Kingdom; cross-sectional	196 patients, 46 smokers, 26 non-smokers (smoking status not recorded for the remaining samples); 33 (19–74), 139 male patients aged at time of first sample, 56 female patients aged 41 (18–84) years at time of first sample, sex of one patient not ascertained; non-smoker patients Amisulpride; psychiatric and neurological disorders	Therapeutic drug monitoring (TDM) of amisulpride from 2002 to the end 2010	Serum concentrations of amisulpride	NA	The mean (95%CI) plasma amisulpride concentration was significantly higher (*t* = 1.99, *p* = 0.02; two-tailed test) in samples from smokers	Poor
de Graan [54], 2012, Netherlands; cohort	276 patients treated with paclitaxel, 62 smokers, 214 non-smokers, 290 patients treated with docetaxel, 75 smokers, 215 non-smokers; Paclitaxel group: 60 (18–82), 140 males, 136 females Docetaxel group: 55 (18–85), 114 male, 176 female; non-smoker patients Docetaxel, Paclitaxel; cancer treatment	Docetaxel-treated patients generally received a 75 to 100 mg/m^2^ dose, depending on tumor type or combination regimen. Paclitaxel-treated patients generally received either 90 mg/m^2^ weekly or 175 mg/m^2^ every 3 weeks	White blood cell (WBC) and absolute neutrophil count (ANC), clearance of Docetaxel and Paclitaxel	CYP 1A2 CYP 1A1 CYP 3A4	Smokers treated with docetaxel showed less grade IV neutropenia than non-smokers. Smokers treated with paclitaxel had less grade III–IV leukopenia than non-smokers and the white blood cell (WBC) nadir was lower in non-smokers than in smokers. Clearance was similar in smokers and non-smokers for both taxanes	Good
Montalli [55], 2012, Brasil; cohort	26, 13 smokers, 13 non-smokers; 22 (± 1.6), 26 males, 0 females; non-smokers Metronidazole; antibiotic	Subjects received a single oral dose of 750 mg of metronidazole	Plasmatic and salivary metronidazole concentrations at 0.5, 1, 1.5, 2, 4, 6, 8, 12, 24 and 48 h after metronidazole administration	CYP 1A2 CYP 1A1 CYP 2E1	Significant reduction in plasmatic metronidazole concentrations in smokers at 1 h, 1.5 h and 2 h compared with non-smokers (*p* < 0.05)	Fair
Unterecker [56], 2012, Germany; cross-sectional	478 psychiatric patients from the University Hospitals of Mainz, Regensburg, and Würzburg, 87 smokers, 140 non-smokers, 251 not known; 49.1 (± 15.5), 174 males, 304 females; non-smoker patients Venlafaxine (VEN), O-desmethylvenlafaxine (ODVEN); psychiatric and neurological disorders	Data of routine therapeutic drug monitoring (TDM) analyses in patients undergoing treatment with Venlafaxine	Serum concentrations of venlafaxine and O-desmethylvenlafaxine	CYP 1A2 CYP 2D6 CYP 3A4 CYP 2C19	In smokers, mean serum levels of ODVEN were 21% lower than in non-smokers. In smokers, the frequency of elevated serum levels was lower than in non-smokers in spite of similar daily doses	Fair
O'Malley [57], 2013, USA; cross-sectional	151 patients, 12 current smokers, 74 current smokers, 63 never smokers; 66 males, 85 females; non-smokers or light smokers Gemcitabine; cancer treatment	All patients have received gemcitabine chemotherapy alone or in combination with oral chemotherapy agents (e.g. erlotinib in a subset of pancreatic cancer patients)	Data on demographics, tumor type, smoking history, and neutropenia throughout the entire treatment course	CYP1A1 CYP1A2 CYP2E1 UDP-glucuronosyl-transferases	Never smokers had increased CTC-AE-graded neutropenia toxicity versus current smokers (odds ratio, OR: 3.5; 95% confidence interval, CI: 1.1–11.4). Among former smokers, higher pack-year histories were associated with lower odds of higher grades of neutropenia toxicity compared to current smokers. Former smokers with < 25 pack-year history had odds of neutropenia toxicity similar to never smokers (OR: 3.4; 95% CI: 1.0–11.7); former smokers with higher pack-year histories (25–49 years and > 49 years) had lower odds of higher toxicity with reference to current smokers (OR: 3.0 and 0.7, respectively). A 5-unit increase in pack-years was found to reduce the odds of having higher hematologic toxicity by 6.3%	Good
Takahashi [58], 2014, USA; cohort	35 healthy volunteers, 17 smokers, 18 non-smokers; non-smokers 26 (± 7), smokers 32 (± 8); 19 males, 16 females; non-smoker volunteers Loxapine; psychiatric and neurological disorders	Subjects were administered inhaled 10 mg dose of loxapine and confined to the clinical research unit under medical observation from the time of check-in procedures until the completion or discharge procedures	For each subject, many parameters were estimated: Cmax, Tmax, Thalf-max, area under the concentration curve (AUC) from 0 to the last measurable value (AUClast), and from 0 to infinity (AUCinf), and T1/2 were estimated for loxapine and 8-OH-loxapine. Clearance uncorrected for bioavailability (CL/F) was estimated for loxapine	CYP 1A2	Loxapine Cmax was similar in smokers and non-smokers. The median loxapine Tmax was 1.88 and 1.01 min for non-smokers and smokers, respectively. Loxapine AUCinf and AUClast values in non-smokers were comparable with smokers. A slight decrease in the observed mean terminal half-life values was observed for smokers (6.52 h for smokers and 7.30 h for non-smokers)	Good
Douglas-Hall [59], 2017, United Kingdom; cross-sectional	187 patients discharged on lamotrigine, 99 smokers, 49 non-smokers, 39 not known; 46.6 (± 17.4), 122 males, 65 females; non-smoker patients Lamotrigine; psychiatric and neurological disorders	Patients discharged on lamotrigine between October 2007 and September 2012 were identified from the pharmacy electronic dispensing database system (JAC)	Determination of the corresponding lamotrigine plasma concentrations and the factors that may affect these	UGT 2B7	Smoking status had no significant effect on dose or plasma levels	Good
Li [60], 2018, USA; cohort	28 healthy patients, 14 smokers, 14 non-smokers; smokers: 51,9 (40–67), 14 males non-smokers: 50,4 (40–66), 14 males; non-smoker patients Pomalidomide; cancer treatment	All patients orally received a 200 mg caffeine capsule on day 6 of each cohort, and on day 8, patients received a single oral 4 mg dose of pomalidomide. All patients were on a methylxanthine-free diet	Pomalidomide plasma pharmacokinetic parameters such as Cmax (maximum plasma drug concentration), Tmax (time when maximum drug concentration is found in the bloodstream), AUC0-inf (area under the plasma concentration–time curve from time 0 to infinity), t1/2 (half-life), CL/F (apparent total plasma clearance), Vz/F (apparent volume of distribution during the terminal phase when dosed orally)	CYP 1A2 CYP 3A4	The Cmax of pomalidomide was 14.4% higher in smokers than that in non-smokers, whereas the AUC0-inf of pomalidomide was 32.3% lower in smokers than in non-smokers. Thet1/2of pomalidomide in smokers was less than in non-smokers (4.8 and 7.8 h, respectively). The mean CL/F of pomalidomide in smokers was greater than that in non-smokers (8.6 and 5.8 L/h, respectively)	Fair
Augustin [61], 2019, Germany; cross-sectional	125 psychiatric patients, 86 smokers, 39 non-smokers; 74 males, 51 females; non-smoker psychiatric patients Clozapine; psychiatric and neurological disorders	Two groups of patients receiving clozapine monotherapy consisting of non-smokers (VNS, *n* = 28) and smokers (VS, *n* = 43) and two groups receiving clozapine with fluvoxamine augmentation consisting of non-smokers (VNS + F, *n* = 11) and smokers (VS + F, *n* = 43)	Serum concentrations of CLZ and NCLZ were compared between the four groups. Dose-adjusted plasma concentrations (ratio of the drug concentration C and the applied daily dose D, C/D, in [ng/mL]/[mg/day]) for clozapine, N-desmethylclozapine as well as the metabolite-to-parent ratio (MPR; NCLZ/CLZ) were calculated	CYP 1A2 CYP 1A1 CYP 2E1	The Clozapine monotherapy smoking group showed lower values of C/D CLZ of 38.6%, C/D NCLZ 35.6% and a higher MPR than in the non-smoking group. The combination of CLZ and fluvoxamine in non-smoking patients led to higher C/D values: C/D CLZ + 117.9%, C/D NCLZ + 60.8% while the MPR did not differ between groups. Changes were comparable to fluvoxamine augmentation in the smoking group with increased C/D CLZ of + 120.1%, C/D NCLZ of + 85.8% and lower MPR	Good
Scherf-Clavel [27], 2019, Germany; cohort	503 patients, 163 smokers, 340 non-smokers; 47.12 (± 12.29), 218 males, 285 females; non-smokers Amitriptyline and nortriptyline, doxepin and nordoxepin, mirtazapine, venlafaxine, clozapine, quetiapine, and risperidone; psychiatric and neurological disorders	Serum concentrations of drugs and their metabolites were determined between January 2009 and December 2010. Dose-corrected, steady-state serum concentrations of individual patients were analyzed retrospectively by linear regression including age, sex, and smoking for the selected pharmaceuticals	Serum concentrations of the selected pharmaceuticals	CYP 1A2 CYP 3A4 CYP 2C19 UGT 1A3 UGT 1A4	Serum levels of amitriptyline (*p* = 0.038), clozapine (*p* = 0.02), and mirtazapine (*p* = 0.002) were significantly lower in smokers compared with non-smokers after correction for age and sex. The ratios of nortriptyline/amitriptyline (*p* = 0.001) and nordoxepin/doxepin (*p* = 0.014) were significantly higher in smokers compared with non-smokers	Fair
Scherf-Clavel [62], 2019, Germany; cross-sectional	124 patients in the Department of Psychiatry, Psychosomatics and Psychotherapy of the University Hospital of Würzburg 36 smokers, 88 non-smokers; 50 ± 21, 38 males, 86 females; non-smokers patients Escitalopram; psychiatric and neurological disorders	Serum concentrations of escitalopram from inpatients and outpatients were determined between 2015 and 2018 in the Department of Psychiatry, Psychosomatics and Psychotherapy of the University Hospital of Würzburg during routine therapeutic drug monitoring (TDM)	Serum concentrations of escitalopram	CYP 1A2 CYP 3A4 CYP 2C19	Smokers received by mean 17.6% higher doses of escitalopram (*p* = 0.026) but showed 31.9% lower serum concentrations (*p* = 0.031). To control for confounders, linear regression analysis showed that dose (*p* < 0.001), sex (*p* = 0.03), and smoking tobacco (*p* = 0.027) did significantly influence serum concentrations of escitalopram with higher levels in women and non-smokers	Good
Turner [63], 2019, United Kingdom; cohort	571 patients 1 month after hospitalization for a non-ST elevation acute coronary syndrome, 157 smokers, 414 non-smokers; 63.5 (± 11.5), 443 males, 128 females; non-smoker patients Atorvastatin; cardiovascular	Participants treated with 80 mg ATV daily at both baseline and V2, or on ATV 40 mg at both baseline and V2, and both drug adherence data and a stored plasma EDTA sample available at V2	Plasma levels of atorvastatin (ATV) and its main acid and lactone metabolites	CYP 1A2 CYP 1A1 CYP 2E1 UGT 1A3 UGT 1A1	Smoking was newly associated with increased ATV lactonization and reduced hydroxylation	Good
Chirehwa [64], 2020, South Africa; cohort	132 patients with rifampicin-resistant TB, 59 smokers, 73 non-smokers; 35.7 (18.8–68.9), 78 males, 54 females; non-smoker patients Cycloserine; antibiotic	A one-compartment disposition model with two clearance pathways, nonrenal (0.35 L/h) and renal (0.43 L/h), described cycloserine pharmacokinetics well	Nonrenal clearance and the volume of distribution	NA	Nonrenal clearance was 41% higher among current smokers than among non-smokers	Good
Kuzin [65], 2020, Switzerland; cross-sectional	611 psychiatric patients, 576 patients under clozapine with co-medication without known effects on cytochrome P450 isoenzymes (326 smokers and 250 non-smokers) and 35 clozapine-medicated patients receiving a co-medication including sertraline (17 smokers and 18 non-smokers); smokers treated with clozapine: 38 (19–72), 26,4% females non-smokers treated with clozapine: 44 (19–88), 42,0% females smokers treated with clozapine and sertraline: 45 (24–58), 41,2% females non-smokers treated with clozapine and sertraline: 43 (21–76), 44,4% females; non-smokers Clozapine and sertraline; Psychiatric and neurological disorders	A large TDM database as part of KONBES, a web-based laboratory information management system for TDM laboratories, consisting of plasma concentrations of CLZ of 1644 inpatients and outpatients (exception: organic mental disorders) was analyzed. Data collection took place between 2005 and 2018 as part of the clinical routine in different institutions as part of the AGATE (AGATE is a cooperation for drug safety in the treatment of psychiatric diseases)	CLZ blood concentrations (in ng/mL) and the plasma concentrations corrected by the daily dose, the so called “concentration-by-dose,” C/D, (in [ng/mL]/[mg/day])	CYP 1A2 CYP 2C19 CYP 3A4 CYP 2D6 CYP 2C9	When comparing smokers and non-smokers in the whole sample (CLZS + CLZ-SERTS vs. CLZNS + CLZ-SERTNS), CLZ blood concentrations and C/D ratios were lower in smokers compared with non-smokers (*p* < 0.001 for both for M-W U), while the applied daily doses were higher in smokers (*p* < 0.001 for M-W U)	Good
Murtadha [66], 2021, Egypt; cohort	36 volunteers, 12 non-smokers, 12 smokers, 12 cannabis smokers; 36 males, 0 females; non-smoker patients Sildenafil; PDE5 inhibitor	All patients received single dose of Sildenafil (50 mg tablet)	Peak concentration of sildenafil in plasma Cmax, the area under the plasma concentration time curve from zero to time (AUC0–t), the time to peak plasma concentration (Tmax), elimination half-life in plasma (t1⁄2) and the area under the plasma concentration time curve from zero to infinity (AUC0–inf) were calculated	CYP 3A4 CYP 2C9	Exposure of sildenafil (AUC0–t) significantly increased in cigarette smokers by 61% (*p* < 0.05). Moreover, the Cmax of sildenafil increased by 63% (*p* < 0.05) in cigarette smokers. The least significant difference pairwise comparison showed a significant difference in the AUC0–t between cigarette smokers and non-smokers (*p* = 0.03). Regarding the AUC0–inf, there was a significant difference between cigarette smokers and non-smokers. The Tmax was comparable among the three groups of the study	Good
Welty [67], 2021, USA; cohort	33 patients, 7 smokers, 26 non-smokers; 44,1 (± 12,5), 11 males, 22 females; non-smokers Lamotrigine; psychiatric and neurological disorders	They collected prospective data from enrolled patients on their use of cigarettes as part for a crossover replication study of generic LTG prducts (Equigen)	Pharmacokinetic characteristics of lamotrigine	UGT1A4 UGT1A1 UGT1A3 UGT1A6 UGT1A7 UGT2B7	Higher cigarette use did not result in a significant change in AUC or Cmax	Fair
Zang [68], 2021, China; cross-sectional	354 Chinese adult psychiatric patients, 105 smokers; 40.2 (± 15.1), 239 males, 115 females; non-smokers Olanzapine; psychiatric and neurological disorders	Prior olanzapine population pharmacokinetic (PPK) models have focused on the effects of sex and smoking on olanzapine clearance	Serum concentrations of olanzapine	CYP 1A2 UGT 1A4 CYP 2D6 CYP 3A4	Olanzapine clearance was increased 1.23-fold by smoking	Good
Sangüesa [69], 2022, Spain; cross-sectional	61 psychiatric inpatients, 37 smokers, 24 non-smokers; 44,18 (± 15,14), 40 males, 21 females; non-smokers Clozapine and Valproic acid; psychiatric and neurological disorders	Routine TDM data collection on inpatients of the Neuropsychiatric Centre Nuestra Senora ˜ del Carmen-Hermanas Hospitalarias (Zaragoza, Spain) treated with Clozapine or both Clozapine plus Valproic acid from January 2018 to September 2021	CLZ (clozapine) and NCLZ (norclozapine) plasma levels, CLZ/NCLZ ratio	CYP 1A2 CYP 2D6 CYP 3A4 CYP 2C19	Smoking habits have an influence on CLZ pharmacokinetic parameters because of an induction of CYP1A2, the main pathway of metabolism of CLZ, that produces a reduction of CLZ and NCLZ plasma level. VPA could be the cause of increasing the CLZ/NCLZ ratio. Independently of the smoking status, the ratio in patients with VPA was increased. The influence of VPA appears to be greater than tobacco induction on these parameters	Good
Moortyhy [70], 2023, USA; non-randomized clinical trial	28 healthy subjects, 14 smokers, 14 non-smokers; 36,4 (± 9,8), 27 males, 1 female; non-smokers; AZD4635; potential anticancer therapy	2 treatment periods: the first (lasting 5 days) involved the administration of a single dose of 25-mg AZD4635 capsules after a 10-h overnight fast; the second period, following the first, lasting 10 days), involved a daily administration of 25-mg AZD4635 capsules after a 10-h overnight fast. On day 6 of period 2 (day 11 of the total study), participants received a single dose of 25-mg AZD4635 capsules concomitant with daily administration of fluvoxamine	Plasma levels of AZD4635, its pharmacokinetics (AUCinf, AUC0-t, Cmax, Tmax and t1/2λz) and the active metabolites SSP-005174X. The ratio of AUCinf to Cmax was also assessed between smokers and non-smokers	CYP 1A2	Co-administration of AZD4635 with fluvoxamine, compared to AZD4635 alone, increased both AUCinf and Cmax and increased the half-life in both smokers and non-smokers, so smoking had no effect on drug metabolism unlike fluvoxamine	Poor

The articles were published from 2001 to 2023 and, among them, 21 were conducted in Europe, 12 in America, 2 in Asia, and 2 in Africa. The effect of smoking on clozapine was reported in 8 studies. Some studies have measured the plasma concentration of the pharmaceutical and the ratio of the plasma concentration to the administered dose. The results consistently indicated that smokers achieved a lower plasma concentration of the drug compared to non-smokers at the same dose, or a lower concentration/dose (C/D) ratio in smokers [28, 43, 61, 70]. In one study, just 4.2% of smokers had a clozapine plasma level greater than 1000 μg/L, the threshold beyond which the risk of convulsive seizures increases. However, after a 6-month period of cigarette smoking abstinence, despite the reduction in the administered pharmaceutical dose, the percentage increased to 41.7% [51].

Another study highlighted a significant increase in the clearance of orally administered clozapine in smokers respect to non-smokers [50]. Besides, the results of another research evidenced that the coefficient of variation of the plasma concentration of clozapine was higher in heavy smokers compared to non-heavy smokers [41]. Similar results were shown in six studies evaluating the effects of smoking on olanzapine. In particular, Patel et al. [52] demonstrated a reduction of the plasma concentration of olanzapine in the smoking group and Zang et al. [69] reported an increase in drug clearance in smokers. For olanzapine, smoking was also responsible for reducing the ratio between plasma concentration and the administered drug dose [39, 43, 45]. In addition, Carrillo et al. [36] highlighted that, after 15 days of antipsychotic therapy with olanzapine, the score of symptoms, assessed using the Brief Psychiatric Rating Scale (BPRS), decreases respect to the score assessed in the drug-free period. Besides, the score was lower for smokers than for non-smokers, indicating that the therapy was less effective in lowering symptoms in smokers [36]. Lower plasma levels in smokers have also been documented for the following pharmaceuticals: mirtazapine, amitriptyline, escitalopram [62], and venlafaxine [56]. Conversely, higher plasma concentrations of amisulpride were found in smokers than in non-smokers [53]. As regards lamotrigine and loxapine, no significant differences in plasma concentrations were recovered between smokers and non-smokers [58, 59, 67]. One study examined the use of zolpidem and any significant variations emerged between smokers and non-smokers [38].

Other studies included in the review demonstrated interactions between cigarette smoking and antineoplastic drugs. For example, smokers undergoing erlotinib treatment presented a two-thirds reduction in the maximum plasma concentration of the drug compared to non-smokers [42]. For the anticancer drugs docetaxel, paclitaxel [54], gemcitabine [57], and irinotecan [47], the reduction in the plasma concentration of the drug in smokers was associated with a lower degree of toxicity for the development of neutropenia compared to non-smokers. Smoking, as an inducer of CYP1A2, has also been found to reduce the bioavailability of pomalidomide [60].

However, in both a study involving imatinib [48] and a study involving a new experimental antineoplastic drug, AZD4635 [70], no significant difference in drug effects was found between smokers and non-smokers.

Regarding pharmaceuticals acting on the circulatory system, being a smoker has led to a reduction in pharmacokinetic indices, such as maximum plasma concentration, half-life, and area under the curve for clopidogrel [49] and verapamil [37], as well as an increase in the metabolism of atorvastatin [63]. No differences between smokers and non-smokers were found on warfarin therapy [44].

Concerning antibiotics, studies found a reduction in plasma concentration of metronidazole [55] and an increase in non-renal clearance of cycloserine [64] in smokers compared to non-smokers. An increase in urinary excretion was observed for the local anesthetic ropivacaine in smokers respect to non-smokers [35]. Other drugs on which smoking can act by reducing plasma concentrations are tizanidine [40] and cilomilast [46].

In contrast to all previous studies, a single study demonstrated significant increased plasma levels of the drug (sildenafil) in smokers compared to non-smokers [66].

## Discussion

Scientific evidence demonstrated that drug metabolism in the human body can be influenced by the exposure to several chemical environmental contaminants. For example, the exposure to PAHs present in cigarette smoke seems to affect the expression of various CYPs, including CYP1A1/2, CYP1B1/2 and CYP2E1, phase II enzymes, transport proteins, plasma protein levels, and liver mass [24, 71]. Similarly, cigarette smoke contains other numerous chemicals, such as acetone, pyridine, heavy metals, benzene, carbon monoxide, and nicotine, that can affect drugs metabolism [24] and the present systematic review was performed to examine the effect of smoking on pharmacokinetic and pharmacodynamics of all type of drugs.

The first relevant finding is that the results of the studies included in the review agree that cigarette smoking influences the kinetics of the drugs taken in therapy. Regarding specific pharmaceutical classes, most included research evaluated the impact of smoking on the kinetics of drugs used in the treatment of psychiatric or neurological patients, highlighting a decrease in the plasma concentration of the drug in smokers respect to non-smokers. The main pharmacokinetic indices altered in smokers compared to non-smokers indicated that the effective concentration of the drug at plasma level was achieved lower in smokers than in non-smokers at the same dose administered and, in general, a reduction in the body’s exposure to the drug. The primary mechanism questioned to explain these findings is the induction that smoking might have on the activity of liver enzymes, particularly CYP1A2, which is responsible for the metabolism of these medicines at the liver level [15, 19]. From a clinical point of view, the increased hepatic metabolism and the consequent reduction in plasma concentrations may lead to a decrease in the therapeutic effect of the drug, as evidenced in patients treated with olanzapine, where smokers showed a more modest reduction in psychiatric symptoms after 15 days of treatment [36]. Besides, the number of cigarettes consumed was also associated with a greater variation in clozapine plasma levels in heavy smokers compared to non-heavy smokers [41]. Therefore, it is crucial to evaluate the degree of exposure to cigarette smoke, especially in psychiatric patients treated with antipsychotics whose metabolism may be influenced by smoking, who are usually subject to a greater degree of dependence.

Also, for antineoplastic drugs, the results of the review indicate a reduction in the effective concentration and a reduction in the body’s exposure to the drug in smokers, involving the induction of various hepatic enzymes of the cytochrome P450 family: CYP1A1, CYP1A2 and CYP2E1 [15, 19, 24–27]. It is relevant to note that, in three studies [47, 54, 57], a reduction in neutropenia was observed in smokers, suggesting a reduced toxicity on blood cells, which could also lead to a reduced toxicity on neoplastic cells, with a consequent reduction in the anti-tumor effect of the drug, increasing the probability of failure of therapy.

Most of the studies were conducted on psychiatric and antineoplastic drugs, but the increase in the metabolism of drugs that act on the cardiovascular system, antibiotics, local anesthetics, drugs acting on the musculoskeletal system, and drugs used in obstructive pulmonary diseases was also highlighted and the theorized mechanisms include the induction or the inhibition of the enzymatic isoforms of cytochrome P450 [35, 40, 46, 55, 64, 66].

Cigarette smoking significantly affects several drug metabolizing enzymes and transporters, in addition to the well-known effects on cytochrome P450 (especially the CYP1A2 isoform). In addition to CYP1A2, smoking also induces CYP2E1, which is involved in the metabolism of alcohol and drugs such as paracetamol, increasing the formation of toxic metabolites [15]. From a clinical perspective, smoking may lead to reduced therapeutic efficacy, requiring higher dosages to maintain effective plasma concentrations, or toxicity to drugs whose metabolism is inhibited or altered. A practical example is the need for higher doses of olanzapine in smokers to compensate for CYP1A2 induction [72]. The role of CYP1A2 induction by smoking is well documented, while the ability of smoking to alter the metabolism of drugs eliminated through other enzymatic and non-enzymatic pathways warrants further exploration. For instance, glucuronidation, a phase II metabolic process catalyzed by UDP-glucuronosyltransferase (UGT) enzymes, appears to be influenced by smoking, as evidenced by the findings related to atorvastatin metabolism. This suggests that smoking may enhance UGT activity, leading to increased drug clearance through glucuronidation. In particular, atorvastatin, primarily metabolized via the CYP3A enzyme, presents a notable case of altered metabolism in smokers. Turner’s study [63] describes an increase in lactone formation, a metabolic product of atorvastatin, suggesting that glucuronidation is upregulated in the presence of smoking. This enhanced biotransformation highlights the potential for smoking to induce glucuronidation pathways, leading to altered pharmacokinetics of atorvastatin. Enhanced glucuronidation may lead to an increased conversion of atorvastatin to its lactone form, potentially altering its pharmacological activity and therapeutic outcomes. This raises important considerations for dose adjustments and monitoring in smokers to whom atorvastatin has been prescribed [63]. Besides, several therapeutic agents rely on glucuronidation as a major pathway for their elimination. The pharmacokinetics of drugs such as lamotrigine, morphine, and certain non-steroidal anti-inflammatory drugs could be altered in smokers if glucuronidation activity is enhanced. However, this pathway should be still studied in depth. Besides, more in-depth studies are needed to explore the effects of smoking on other enzymes, such as CYP3A4, and on renal transporters, as well as the possibility of monitoring and tailoring drug dosages by phenotypic and genotypic tests (e.g. caffeine test for CYP1A2) [73].

Some xenobiotics, such as PAHs, produced by tobacco combustion, induce CYP1A1, CYP1A2, CYP1B1, CYP2A8 and UGT genes through transcriptional mechanism. These xenobiotics act as a ligand for the aryl hydrocarbon receptor (AhR). When binding a ligand, the receptor is activated to translocate into nuclei and, during the nuclear translocation process, dissociates from the 90 kDa heat shock protein (Hsp90) to form a heterodimer with Arnt (Ah receptor nuclear translocator). The heterodimer complex binds a DNA response element called xenobiotic responsive element (XRE), located upstream of the target genes of many drug-metabolic enzymes to activate their transcription [74].

With regard to P-gp, the most common mechanism hypothesized for its inhibition by the toxic substances present in cigarette smoke is the competition with export substrates. P-gp dysfunction could be associated with reduced cell viability [30].

This systematic review showed a statistically significant reduction in plasma levels of the following drugs in smokers compared to non-smokers: olanzapine [72], clozapine [27], quietapine [27], risperidone [27] (antipsychotics), escitalopram [62], amitriptyline [27], doxepin [27], mirtazapine [27] (antidepressants), verapamil [37] (antihypertensive), metronidazole [55] (antibiotic), irinotecan [47], and erlotinib [42] (antineoplastics). With regard to antipsychotics and antidepressants, the main metabolizing enzymes induced by smoking were found to be mainly the CYP1A2 and CYP2C19 isoforms, according to the transcriptional mechanism illustrated above [74]. Therefore, in order to provide individualized therapy, physicians should inform patients about the interactions between smoking and metabolism of these drugs and consider smoking status in the development of the treatment plan, evaluating an increase in drug dosage. The same transcriptional mechanism of the CYP1A2 isoform has been shown for verapamil [37]. The latter is responsible for the reduction in the plasma concentration of metronidazole in smokers as a result of the transcriptional mechanism of the CYP1A2 isoform; therefore, in this case too, physicians should optimize the dosages of this antibiotic by taking into account the length of time that its concentration exceeds MIC (Minimum Inhibitory Concentration) values [55]. For antineoplastics, the reduction in plasma concentration is linked to the induction of the CYP1A2 isoform by smoking by the same mechanism. In both cases, this condition is associated with the risk of treatment inefficacy [42, 47]. To overcome this problem, some studies evaluated the possibility to use higher doses of erlotinib in smoker patients with non-small-cell lung cancer. Given this evidence for current smokers, the evaluation of the appropriate dose should also be considered for patients exposed to environmental tobacco smoke [42]. The opposite situation occurs for sildenafil, which showed increased plasma levels in smokers compared to non-smokers [66]. Probably, the mechanism behind this phenomenon is the downregulation of the CYP3A4 isoform, which is mainly responsible for the metabolism of this drug. The inhibitory effect of smoking on CYP3A4 is linked to an increase in IL-6, the expression of which is stimulated by the nicotine present in cigarette smoke [66]. Thus, cigarette smoking increases the bioavailability of sildenafil without altering its pharmacodynamic, safety or tolerability profiles. In light of this, the risk–benefit assessment in smokers suggests the use of a lower starting dose than in non-smokers (25 mg instead of 50 mg), with a further dose increase if clinical efficacy is not achieved [66].

In contrast to the CYP1A2 and CYP2C19 isoforms, for which the inducing effect of cigarette smoking has been extensively studied and demonstrated, CYP3A4 continues to be a controversial topic. Indeed, the latter was found to be induced by smoking in one study included in our review [27], while it was inhibited in another [66].

The present systematic review has some limitations. First, the results of the included studies are heterogeneous and did not allow to perform a meta-analysis. Besides, even if the results of all the research evidence pharmacokinetic interactions between smoking and drugs, given the huge number of chemicals present in cigarette smoke and capable of interacting with biological receptors, pharmacodynamic interactions cannot be ruled out. As a result, we cannot exclude the possibility that smoking’s action may interfere with the metabolism of other classes of drugs than those studied in this review, and through complex mechanisms, including those of a pharmacodynamic nature. Therefore, additional epidemiological studies should be conducted to increase knowledge on interactions between smoke and drug metabolism. Finally, we did not include the specific term “electronic cigarette” in the search string, but we used the general term “smoking.” We did not recover any article on electronic cigarettes by the use of our search strategy. Given the possible differences in the effect of traditional and electronic cigarettes on the drugs metabolism, in future it should be interesting to explore this issue.

## Conclusions

Overall, the results of the present review indicate a potential increase in the risk of therapeutic failure for smokers assuming drugs whose metabolism may be influenced by smoking, and represent a further strong motivation to encourage interventions to stop smoking cigarettes. If the goal of smoking cessation is not achieved, smoking patients require greater attention in formulating a personalized therapy, monitoring, and modifying dosages, compared to non-smokers, to be re-evaluated over time if they were to stop smoking. Furthermore, an in-depth understanding of the relationships between cigarette smoking and drugs is fundamental to improve therapeutic outcomes, an aim to be pursued also by increasing research through new studies performed for evaluating differences in the clinical effect of therapy between smokers and non-smokers.

## Data Availability

Data are provided as tables and figures directly within the manuscript, and raw data are available via e-mail upon request to the corresponding author.
